# Performance Comparison of Machine Learning Models for Concrete Compressive Strength Prediction

**DOI:** 10.3390/ma17092075

**Published:** 2024-04-28

**Authors:** Amit Kumar Sah, Yao-Ming Hong

**Affiliations:** Nanhua University, Chiayi 62248, Taiwan

**Keywords:** concrete compressive strength, regression tree, artificial neural network (ANN), root mean square error, coefficient of correlation

## Abstract

This study explores the prediction of concrete compressive strength using machine learning models, aiming to overcome the time-consuming and complex nature of conventional methods. Four models—an artificial neural network (ANN), a multiple linear regression, a support vector machine, and a regression tree—are employed and compared for performance, using evaluation metrics such as mean absolute deviation, root mean square error, coefficient of correlation, and mean absolute percentage error. After preprocessing 1030 samples, the dataset is split into two subsets: 70% for training and 30% for testing. The ANN model, further divided into training, validation (15%), and testing (15%), outperforms others in accuracy and efficiency. This outcome streamlines compressive strength determination in the construction industry, saving time and simplifying the process.

## 1. Introduction

Concrete is one of the most crucial and ancient construction materials used in the construction industry [1]. The primary raw materials for concrete construction include cement, water, sand, and aggregates. Additionally, certain chemicals or admixtures are incorporated to enhance the initial or final properties of concrete. Globally, around 3 billion tons of raw materials are utilized annually for concrete production [2,3]. However, selecting the appropriate ingredients in the right quantities remains a challenging task for producers aiming to achieve specific concrete strength.

Concrete, being a versatile material adaptable to various requirements and forms, plays a pivotal role in meeting diverse construction needs. Ensuring the desired strength of concrete is critical for safety [4] as failure to achieve the required strength may result in construction failures, leading to potential loss of life and resources. Traditional strength assessment methods involve complex, time-consuming compression tests under specific laboratory conditions for 28 days [5], and they are susceptible to experimental errors. If concrete fails the test, there is limited scope for adjustments, necessitating a complete restart and prolonged waiting for new results.

Estimating compressive strength before the 28-day mark offers several advantages, including improved efficiency through pre-scheduling of operations. The composition of concrete, its age, water-to-cement ratio, curing conditions, and other factors significantly influence its compressive strength [6]. The relationship between concrete strength and its constituents is highly nonlinear, rendering Abrams’ law insufficient in establishing a dynamic relationship [3].

To address this issue, researchers have explored data-driven models for the evaluation of concrete compressive strength, introducing the concept of machine learning. Machine learning involves training a machine on past data or experiences, enabling it to perform tasks more efficiently than humans. Supervised learning, unsupervised learning, and reinforcement learning are different approaches to machine learning [7]. In supervised learning, the machine learns from labeled data, while unsupervised learning involves unlabeled data. Reinforcement learning occurs when the machine learns from user feedback.

Machine learning models are fed input, which produces output based on applied algorithms [8]. Iterative feedback improves model performance with each repetition. Various algorithms, such as K-means, linear regression, random forest, logistic regression, support vector machine (SVM), naive Bayes, K-nearest neighbors (KNN), decision tree, and artificial neural network (ANN), are commonly used in machine learning. The selection of algorithms depends on data types and result requirements.

This study utilizes ANN, SVM, regression tree (RT), and multiple linear regression (MLR) to predict concrete compressive strength using eight attributes: water, cement, coarse aggregate, blast furnace slag, age, superplasticizer, fly ash, and fine aggregate. By doing so, we aim to provide insights into the effectiveness of these machine learning models in accurately predicting concrete compressive strength, thereby aiding producers and engineers in optimizing concrete mix designs and ensuring structural integrity.

## 2. Methods

To identify the optimal model for predicting future data, various regression methods were employed in this study. Specifically, four distinct approaches were utilized to establish a model for predicting concrete compressive strength based on several dependent variables, such as cement, water, aggregates, and others. The four methods employed in this investigation include artificial neural network, multiple regression analysis, support vector machine, and regression analysis.

### 2.1. Artificial Neural Network (ANN) Model

Artificial neural networks (ANNs) are information-processing systems based on how biological nervous systems, like the brain, process information [9]. An artificial neural network is a network of interconnected nodes, referred to as artificial neurons, designed to emulate the functioning of the human brain [10,11]. These neurons are linked to each other, facilitating the transmission of signals from one to another. Upon receiving a signal, each neuron processes it and generates an output computed by a nonlinear function of the sum of its inputs. Neurons possess weights that dynamically adjust during the learning process to enhance performance. The network comprises different layers, with each layer containing varying numbers of neurons, ranging from tens of thousands to less than ten [12].

The distinctive feature of artificial neural networks, setting them apart from conventional problem-solving methods, lies in their learning capability. The learning process, termed training, allows the network to adapt its problem-solving approach based on the nature of the problem and specific requirements. However, this adaptability also introduces a challenge, as the network’s results can sometimes be unpredictable due to its unique problem-solving approach [13].

For processing with an artificial neural network, available data are initially randomly divided into three sets. The first set is the training set (60 to 75%), followed by the validation (or fine-tuning set, 15 to 20%), and the test set (15 to 20%). These sets are utilized in the training, validation, and testing steps, respectively. Each step serves a specific purpose in the artificial neural network workflow. The training aims to minimize errors, such as mean square error (MSE) or root-mean-square error (RMSE), by adjusting the connections between neurons and weights to produce output results as close as possible to the target values. Validation steps operate independently from the training set, contributing to the refinement of the model. Finally, the testing process assesses the accuracy of the developed model [14].

### 2.2. Multiple Linear Regression Model

A regression model illustrates the relationship between a dependent variable, denoted as y, and an independent variable, denoted as x. When there is only one independent variable, it is referred to as a simple regression model. However, when there are multiple independent variables, the model is termed a multiple regression model. Regression models serve to identify correlations between predictors and criterion variables, aiming to uncover relationships between them [15].

In a simple regression model, the objective is to find the best-fit line that accommodates all the data for the dependent and independent variables. Conversely, a multiple regression model seeks a plane that optimally fits all available data. Models with more than two independent variables introduce complexity, yet they can still be analyzed using various methods. In interactions, the relationship between an independent variable and the dependent variable may be linear, curvilinear, or dependent on the value of another independent variable [16]. Equation (1) outlines the fundamental structure of a multiple linear regression model.

(1) $$y=a_{o}+\sum_{j=1}^{m}a_{j}X_{j}$$

where y represents the output data, *X*_*k*‘s_ are the input variables serving as independent variables, and *a*_0_, *a*_1_, *a*_2_, …, *a*_m_ are the partial regression coefficients. The method of least squares is employed in multiple regression to determine the best-fitting line and regression coefficients [17]. Various software tools, such as Mathematica, Stata, and MATLAB software offer a matrix approach to solving multiple linear regression problems, as demonstrated in studies by [18,19].

### 2.3. Support Vector Machines (SVMs)

Support vector machines (SVMs) are designed to learn from training data and aim to generalize their knowledge to accurately predict outcomes on new, unseen data [20]. SVMs have emerged as a significant tool in pattern recognition and regression, known for their excellent generalization performance [21]. This method is often considered a formidable competitor to artificial neural network (ANN) models, capturing the attention of researchers in both the neural network and programming communities. SVMs have found applications in real-world scenarios and were initially introduced by Vapnik and his colleagues [22]. Capable of addressing both classification and regression problems in supervised machine learning, SVM separates two classes of points using a hyperplane, akin to logistic regression and linear regression.

What sets SVM apart is its unique approach to creating two marginal lines parallel to the hyperplane. These margin lines ensure a certain distance, allowing easy linear separability for both classification points. The parallel margin lines pass through the nearest points from each class to the hyperplane. Therefore, SVM not only focuses on a single hyperplane but creates two parallel hyperplanes. One hyperplane passes through the nearest positive points, and the other passes through the nearest negative points, maximizing the distance between them. This configuration provides the SVM model with a cushion to better divide the two classes, resulting in a more generalized and effective predictive model.

Support vectors are the points that pass through the margin line or plane, which can be one or more. While linearly separable data simplify the creation of the hyperplane, SVM employs a technique called SVM kernel for nonlinearly separable data. The SVM kernel aims to transform 2-D or low-dimensional data into higher dimensions for improved classification by the hyperplane. Various types of kernels are available, such as polynomial, RBF, sigmoid, etc., chosen based on the data and scenario. Among these, RBF is the most famous and mainly used for calculation [23].

This study employs the MATLAB application for the SVM model, utilizing the regression learner add-on app under the machine learning and deep learning section. The process involves importing data, selecting the SVM model, and running the analysis. MATLAB offers different SVM models, including linear, quadratic, cubic, fine Gaussian, medium Gaussian, and coarse Gaussian SVMs. The output is presented in terms of root-mean-square error (RMSE) where the model with the smallest RMSE value indicates better performance and is chosen for further prediction with test data. The simplicity and speed of this entire process make it highly efficient.

### 2.4. Regression Tree (RT)

A decision tree is a tree-like structure where each node represents a test on a given feature or attribute, and the branches coming out of the node indicate the outcome of the test. The nodes with no branches, also known as decision nodes, are called leaf nodes or terminal nodes, holding the class label, which can be a classification or regression value. The top node in the tree structure is termed the root node.

Decision trees can be used for decision-making purposes, to provide a visual and explicit representation of the decision process. They apply to both classification and regression tasks, with regression trees specifically designed for handling continuous data. The root node initially encompasses all the data in the dataset, and based on the test results, the dataset splits, which is known as a splitting action. The process continues with additional tests on subsequent decision nodes until a class or value is assigned to the leaf node. The goal of the regression tree is to find the optimal partitioning of the feature space that minimizes the variance of the target variable within each partition [24].

Despite their intuitive tree-like structure, decision trees can become complex and lengthy in practice, making interpretation challenging. They also perform feature selection or screening of variables and accommodate both categorical and numerical data. Two types of decision trees exist in machine learning algorithms: regression trees, used for continuous outcomes, and classification trees, employed when the outcome falls into a finite number of groups. Together, they are referred to as classification and regression trees (CART). In regression trees, each leaf represents a numeric value, while classification trees yield results like true or false or other distinct categories in their leaves.

Regression trees commonly use impurity measures such as least squares and least absolute deviation [25]. They are adept at handling intricate data structures. This study utilizes MATLAB (R2017a) software for processing regression trees through regression learner add-ons. The software tests three different regression tree models: fine, medium, and coarse. It evaluates each tree’s performance in terms of root-mean-square error (RMSE). The regression tree model with the lowest RMSE is selected for further processing and prediction, making the overall process straightforward and efficient.

### 2.5. Data Description

In pursuit of the objectives outlined in this study, a dataset consisting of 1030 entries, each containing various parameters related to the concrete mix, along with their corresponding compressive strengths, was sourced from the online platform Kaggle. The data were provided by Prof. I-Cheng Yeh from Chung-Hua University in 2007 and can be downloaded from the following link: “https://archive.ics.uci.edu/static/public/165/concrete+compressive+strength.zip (accessed on 4 January 2021)”. Prior to analysis, the collected data underwent a meticulous accuracy check and subsequently underwent a process of data preprocessing using MATLAB software. This initial phase ensures the quality and readiness of the dataset for further analysis and investigation.

#### 2.5.1. Data Preprocessing

Raw data obtained from any source may not be directly suitable for data analysis without undergoing necessary preprocessing steps. This is because the data may contain missing values, erroneous inputs, or formats that are not conducive to analysis. These issues could introduce abnormalities in the output, necessitating careful preprocessing to make the data suitable for use.

In this study, various data preprocessing steps were employed. These included checking for outliers, handling null values, and feature scaling. These operations not only render the data usable but also contribute to increased accuracy of the results. Table 1 presents different statistics related to the parameters utilized in this study, offering insights into the characteristics of the dataset after undergoing the specified preprocessing steps.

#### 2.5.2. Association of Data

(a) Variance

Variance represents the amount of spread in the dataset. It calculates how far each number is present in the dataset from the mean of the whole data. It is an important way to find out the random variable that can be present in the given range. It is calculated by squaring the difference between the mean and each variable and adding them together then dividing the sum by the total number of variables. The calculating formula for finding the variance is as follows: The variance is a statistical measure that quantifies the degree to which data points in a dataset deviate from the mean. It assesses the spread or dispersion of the data. Specifically, variance calculates how far each individual data point is from the mean of the entire dataset. The calculation involves squaring the difference between each data point and the mean, summing these squared differences, and then dividing by the total number of data points.

Mathematically, the formula for calculating the variance (σ) is as follows:

(2) $$the\;\sigma^{2}=\frac{\sum {\left(X-\mu \right)}^{2}}{N}$$

‘X’ represents individual data points from the dataset, while ‘μ’ denotes the mean value calculated across all datasets. ‘N’ stands for the total number of data points in the dataset. A higher variance value suggests that the data points in the dataset are more dispersed from the mean, indicating greater variability. Conversely, a lower variance value suggests that the data points are closer to the mean, indicating less variability. Table 2 presents the variance values for various attributes considered in this study.

(b) Correlation

The correlation coefficient for any pair of attributes within a dataset always falls within the range of −1 to +1. This metric is utilized to gauge the degree of relationship between a given attribute and others present in the dataset.

A negative correlation coefficient indicates that as the value of one attribute increases, the value of another decreases. Conversely, a positive correlation coefficient suggests that an increase in the value of one attribute corresponds to an increase in the value of another. When the correlation coefficient is close to +1, it indicates a stronger correlation between the two attributes. For identical attributes, the correlation coefficient is exactly 1, signifying identical behavior.

Table 3 displays the correlation coefficients between compressive strength and other attributes or variables. A higher correlation coefficient between cement and compressive strength implies a significant role of cement in achieving higher strength. This pattern holds true for various other variables as well.

### 2.6. Evaluation of Findings

Four distinct methods were employed to assess the generalization efficiency of the trained model, aiding in the identification of models with superior performance and accuracy. The first method utilized in this study is the mean absolute deviation (MAD). MAD is calculated by dividing the sum of the absolute differences between the actual and predicted values by the total number of observations. This metric provides insights into the variability within a dataset. A lower MAD value indicates better model performance. The equation for MAD calculation is as follows:

(3) $$\mathrm{MAD}=\frac{\sum_{t=1}^{n}\left|A_{t}-F_{t}\right|}{n}$$

In this study, the unit of MAD will be MPa. The second method is the coefficient of correlation (CC). The formula to calculate the CC is

(4) $$\mathrm{CC}=1-\left[\sum_{1}^{n}{\left\{\frac{A_{t}-F_{t}}{{A_{t}}^{2}}\right\}}^{2}\right]$$

The value of CC should be approaching 1, which will represent the best model.

Another method is root-mean-square error (RMSE), which is the standard deviation of residuals. In other words, it tells how concentrated the data are around the line of best fit.

(5) $$\mathrm{RMSE}=\sqrt{\frac{\sum_{i=1}^{n}{\left(A_{t}-F_{t}\right)}^{2}}{n}}$$

The lower value of RMSE is indicative of good performance. In this study, the unit of RMSE will be MPa.

And the last method is known by the name of mean absolute percentage error (MAPE). It is also a statistical measure of how accurate a forecast system is. It measures accuracy in percentage. It is calculated by the formula:

(6) $$\mathrm{MAPE}=\frac{\sum_{i=1}^{n}\left|\frac{A_{t}-F_{t}}{A_{t}}\right|}{n}\times 100$$

Lower MAPE values show more accuracy of the given method. In all the above four equations, A_t_ is the actual value, F_t_ is the predicted value of our trained model, and n is the size of the dataset.

## 3. Results and Discussion

### 3.1. Artificial Neural Network

In this study on artificial neural network models, eight different input variables were utilized to predict concrete compressive strength using the ANN. When running the ANN model in Matlab, a total of 1030 specimens were randomly divided into two subsets: 70% for training and the remaining 30% equally divided for validation and testing. Various training algorithms were employed to optimize results, including different network types, such as feed-forward backprop, cascade-forward backprop, competitive, Elman backprop, feed-forward distributed time delay, feed-forward time delay, NARX, series-parallel NARX, layer recurrent, and others.

Throughout the process, the input data and target data remained constant. There was flexibility in selecting different training functions, such as TRAINLM, TRAINOSS, TRAINR, TRAINRP, and many others. Additionally, two distinct adaption learning functions, LEARNGD and LEARNGDM, were considered for improved results. Three performance functions, namely, MSE, MSEREG, and SSE, were employed for evaluation.

Crucial factors influencing the ANN model’s performance include the number of neurons and the number of hidden layers. The transfer functions of each layer, such as TANSIG, PURELIN, and LOGSIG, were also tested to assess model performance. Table 4 presents various architectural models used in the process where, for example, ‘8-10-1′ denotes 8 input variables, 10 neurons, and 1 output. Similar notations apply to other architectures, with the first and last numbers representing input and output, respectively, and the numbers in between indicating neurons in the hidden layers. Table 5 outlines the optimum parameters for the best-trained model, providing valuable insights into the configuration that yielded the highest performance.

Here, the 8-15-1 model is selected as the best ANN model as it achieves higher accuracy.

**Figure 1:**
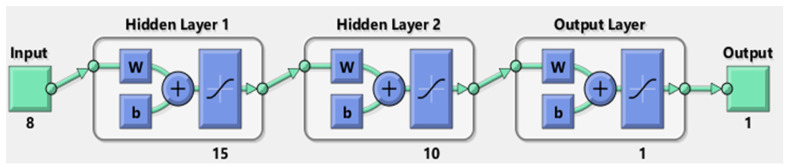
Outline of 8-15-10-1 architecture.

Figure 1 illustrates the architecture of the best-trained model, featuring two hidden layers. The first hidden layer consists of 15 neurons, while the second hidden layer employs 10 neurons. TANSIG transfer function is applied to each hidden layer.

**Figure 2:**
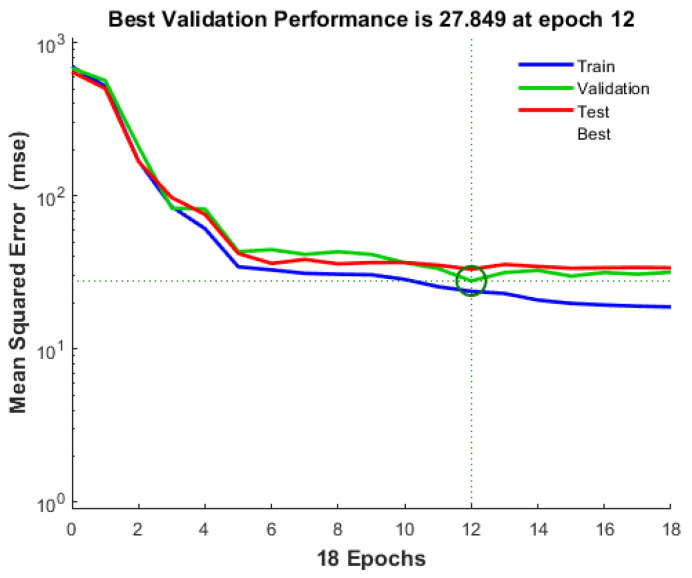
ANN model’s best validation performance.

Figure 2 displays the validation performance throughout the training, validation, and testing steps. This visualization presents the mean square error (MSE) for the best-performing architecture, indicating the network’s learning progress. Notably, at epoch 12, the model achieved its optimal validation performance with the lowest mean square error. This observation suggests that the model continued training until the network error on the validation vectors reached its minimum value. The evaluation process concluded after 6 additional iterations, stopping at epoch 18, immediately following epoch 12 with the best validation performance.

**Figure 3:**
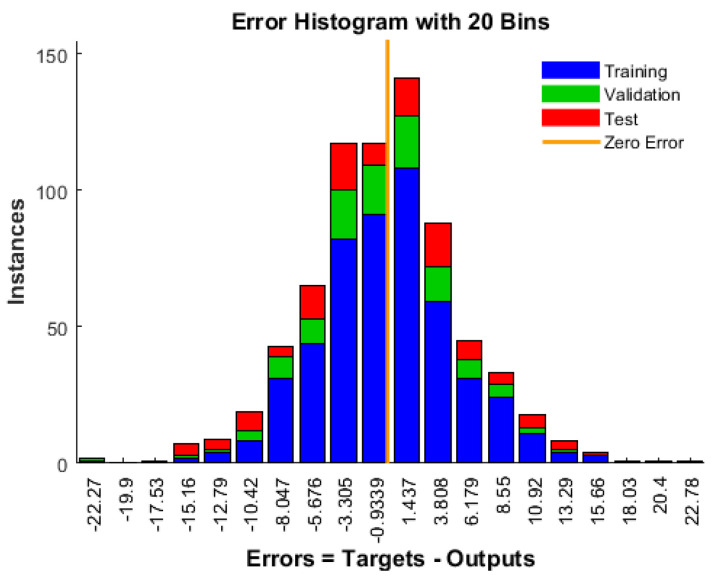
Error histogram for all the steps of ANN.

Figure 3 depicts the error histogram of the best-trained model wherein the error range is segmented into 20 smaller bins. Bins are represented by the number of vertical bars on the graph. The error range on the graph spans from negative 22.27 to positive 22.78. In the graph, the blue bars denote values for training, the green bars represent validation, and the red bars signify test values. The zero error line is also featured on the graph. A higher concentration of bin heights near the zero error line indicates a well-performing model. In this context, an error is calculated as the difference between predicted compressive strength and measured compressive strength.

**Figure 4:**
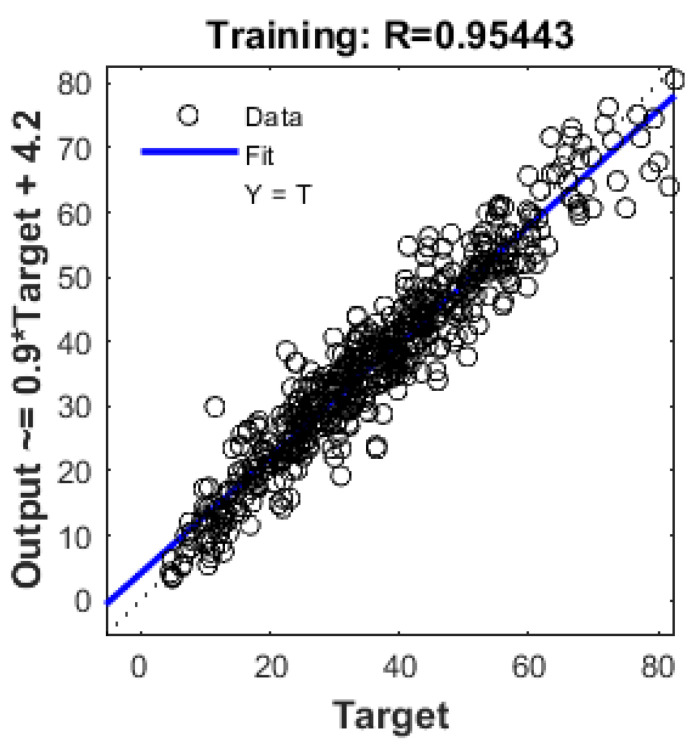
Output Vs target by ANN model for training data.

**Figure 5:**
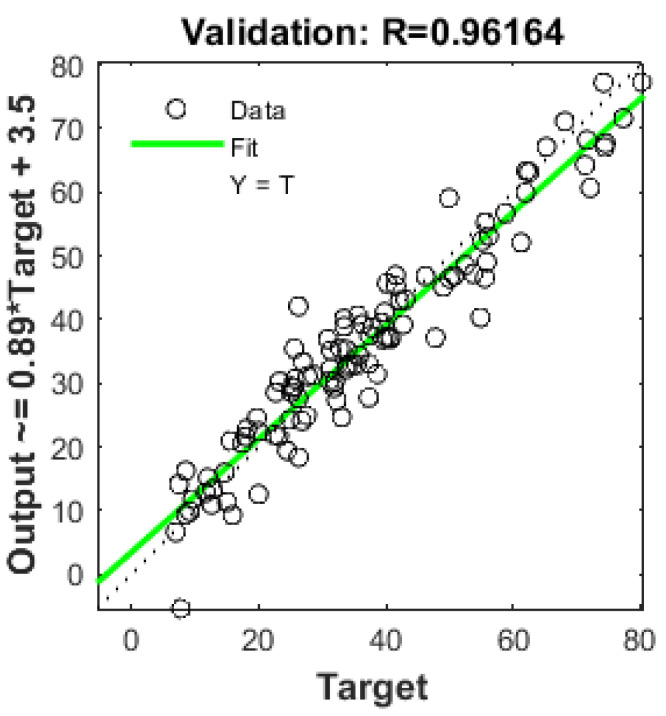
Output Vs target by ANN model for validation data.

Figure 4 and Figure 5 illustrate the relationship between predicted compressive strength and measured compressive strength. In Figure 4, which represents the training steps, the value of R is 0.95443, indicating a favorable result for the model. The same positive representation holds for Figure 5, which showcases the results for validation steps, with an R-value of 0.96164. 

### 3.2. Multiple Linear Regression

This study employed a total of 1030 specimen samples for training and testing the multiple linear regression model. Each specimen sample consists of 8 different input variables representing various attributes of concrete manufacturing. The output variable provides the compressive strength of concrete. For model training, 721 samples (70% of the total) were selected, while the remaining 309 samples (30% of the total) were designated for testing the model’s performance.

During the training step, the weight parameters for different attributes were determined as follows: 0.1194, 0.1074, 0.089, −0.1909, 0.1989, 0.0162, 0.0145, and 0.1229, respectively, for the 8 different attributes. Additionally, the value of α0 was found to be −9.2973.

The multiple linear regression equation is expressed as follows:

Y = (−9.2973) + (0.1194⋅A2) + (0.1074⋅B2) + (0.089⋅C2) + (−0.1909⋅D2) + (0.1989⋅E2) + (0.0162⋅F2) + (0.0145⋅G2) + (0.1229⋅H2)

Here, A2, B2, C2, etc., represent the values of input variables such as cement content, blast furnace slag, fly ash, water content, superplasticizer, coarse aggregate, fine aggregate, and the number of days (age), respectively.

Table 6 displays the performance measurement of the generated model for the test data.

**Figure 6:**
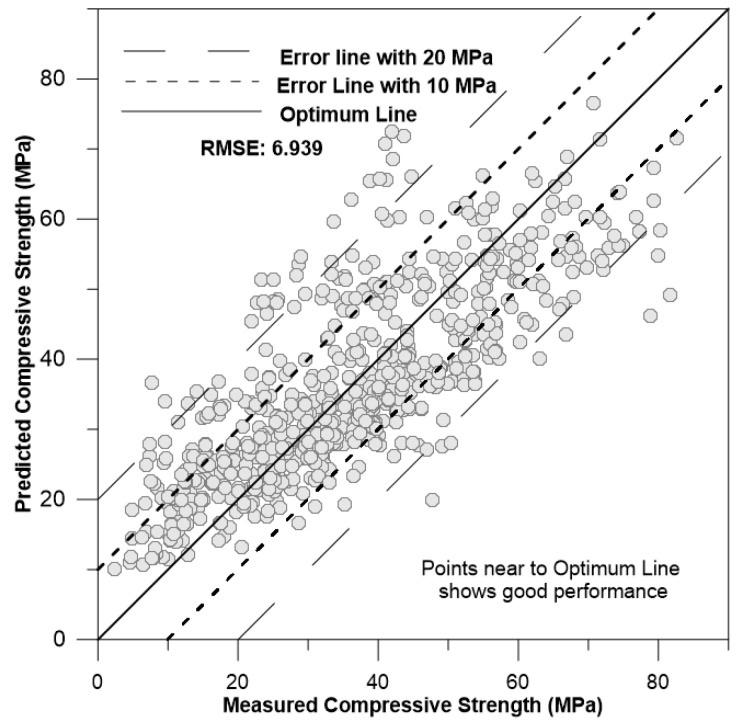
Measured vs. predicted compressive strength.

The graph in Figure 6 shows the measured vs. predicted compressive strength with RMSE = 6.9395.

Figure 6 illustrates two different error lines and an optimum line. The optimum line consists of points where the predicted values equal the measured values of concrete strength. The error lines are situated within a range of ±10 MPa and ±20 MPa on both sides of the optimum line. A higher concentration of points near the optimum line indicates a good performance result. When points fall between the optimum line and the first error line (closer to the optimum line), the model performs well with some errors. If some points extend beyond the first error line, the model exhibits a slightly higher error. In this case, a significant number of points are scattered outside the first error line, and some are even outside the second error line. This suggests that the model is not performing well and is lacking higher accuracy in its predictions.

### 3.3. Support Vector Machine (SVM)

**Figure 7:**
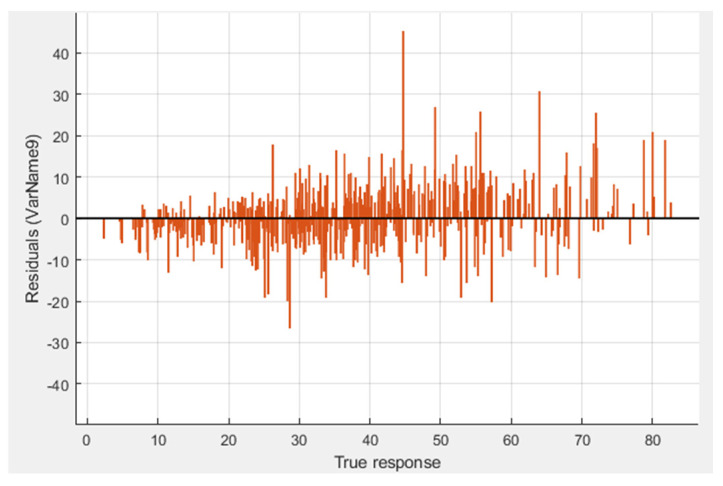
Residual plot of SVM trained model.

The support vector machine (SVM) model employed the same sample data to facilitate a proper comparison of different model performances. The entire dataset was randomly divided into two subsets: 70% for training and 30% for testing. The model training was conducted using the MATLAB regression learner application. Various SVM configurations were explored to achieve higher accuracy. The results of the trained model are depicted in Table 7 where cubic SVM is found to be better than others. This model is exported to the further prediction of future data. Figure 7 shows the residual graph of the true value and the residual value.

### 3.4. Regression Tree

Similarly to the last two models, the regression model also utilized the same set of 1030 data samples divided for training and testing purposes. Each sample data point comprises 8 different input variables and 1 output variable. The various input variables represent parameters for concrete manufacturing, including the weight of cement, different types of aggregate, water, and other crucial factors. The model training was carried out using the MATLAB regression learner application, with the selection of all regression trees to achieve higher accuracy among the available options in MATLAB. The results of the trained model are presented in Table 8.

**Figure 8:**
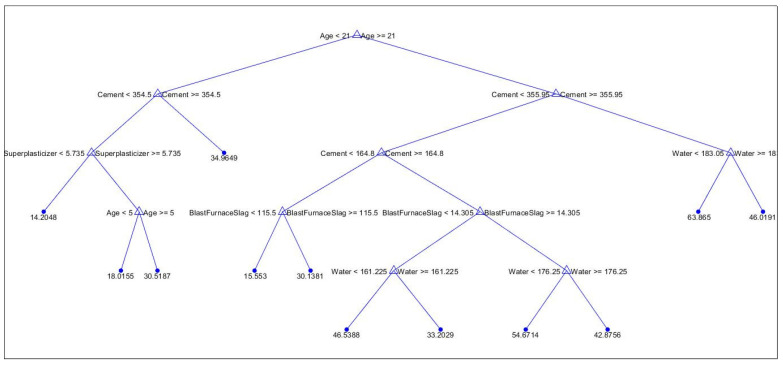
Regression tree structure of the generated model.

Based on the above results, it is observed that the fine tree model outperforms the others. This model has been selected for further predictions on future data, and its decision tree structure is examined for insights. Figure 8 illustrates the regression tree with a pruning level of 94 out of 106.

### 3.5. Performance Comparison

Optimum parameters were determined for all the models tested in this study. For the ANN model, various parameters were altered, including the number of hidden layers, the number of neurons, and the training model. Each change in these parameters led to a corresponding change in the model’s results. Multiple attempts were made using trial and error methods to identify the optimum parameters. Similar steps were undertaken for the other models to find the best fit. Table 9 presents a comparison of the performance measurements for different models.

Upon reviewing the performance results of various models, it becomes evident that the ANN model outperforms the other models as its RMSE and other values are the smallest in the table. The next best-performing model is SVM, which predicts with slightly less accuracy than the ANN model. Conversely, it can be concluded that the multiple regression model performs the worst under these conditions.

**Figure 9:**
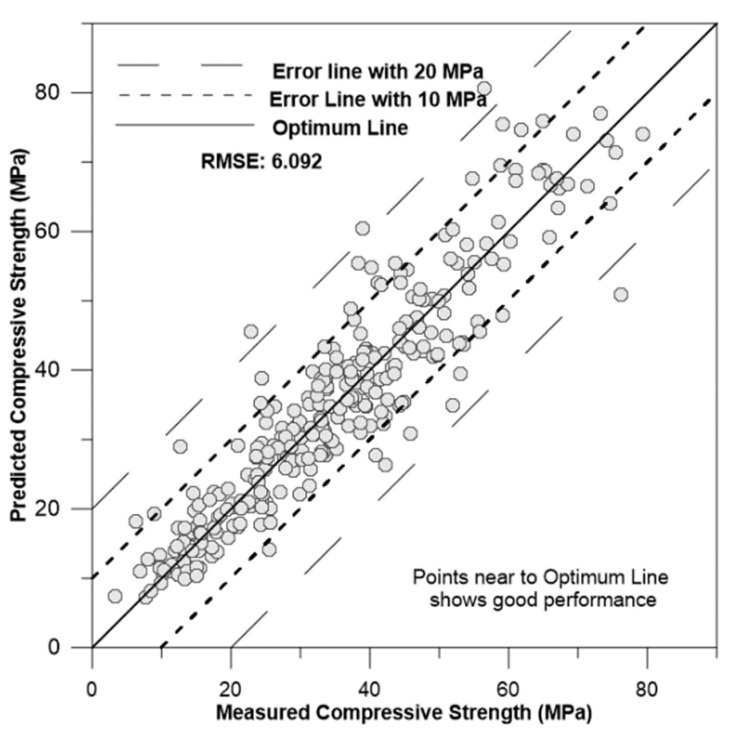
Correlation of ANN-predicted concrete strength to measured concrete strength for simulation data.

**Figure 10:**
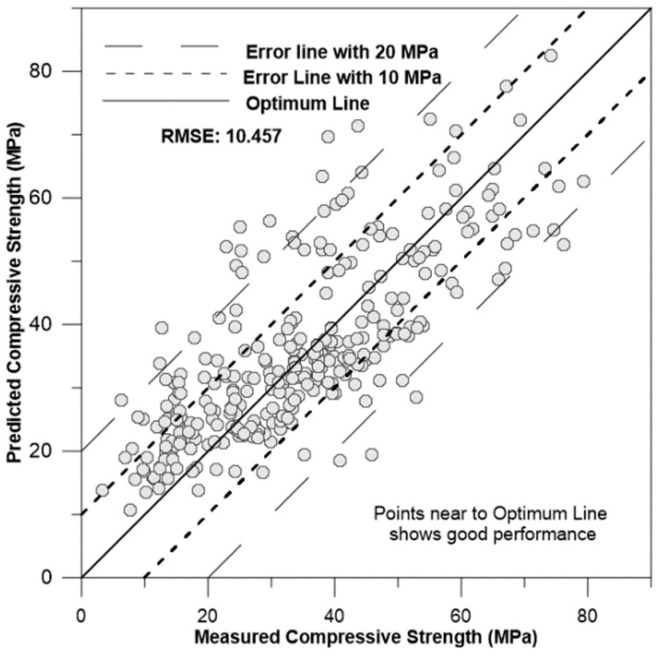
Correlation of MLR-predicted concrete strength to measured concrete strength for simulation data.

**Figure 11:**
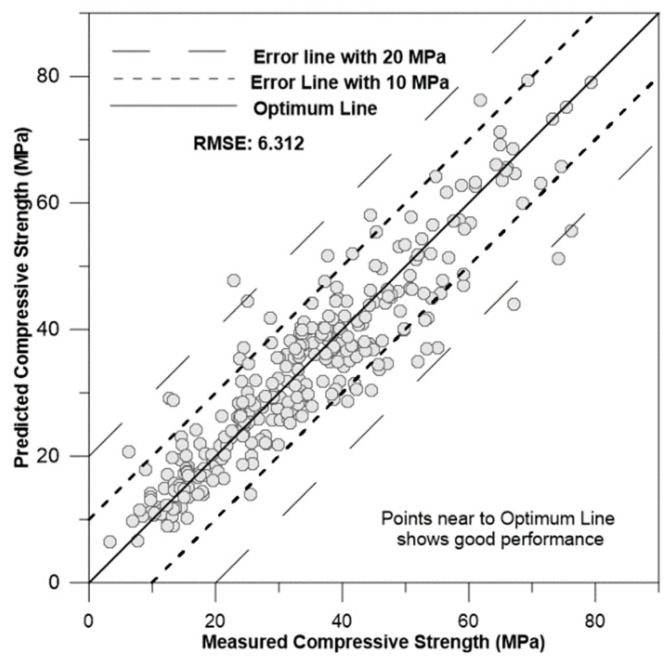
Correlation of SVM-predicted concrete strength to measured concrete strength for simulation data.

**Figure 12:**
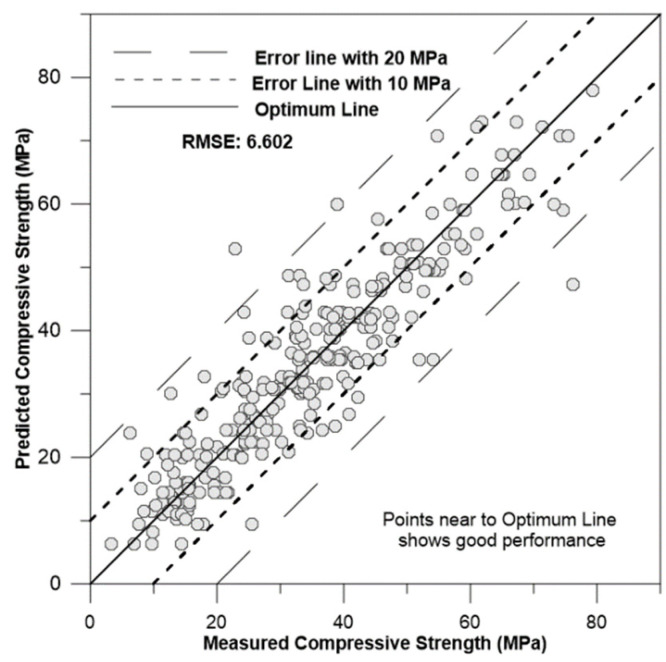
Correlation of RT-predicted concrete strength to measured concrete strength for simulation data.

Figure 9, Figure 10, Figure 11 and Figure 12 depict the correlation of predicted concrete strength by different models to the measured concrete strength for simulated data.

From the above four figures, it can be seen that points in the ANN-predicted concrete strength to measured concrete strength are closer to the optimum line in Figure 9. Additionally, these points mostly fall within the error margin lines of 10 MPa and 20 MPa. This proximity demonstrates the superior performance of the ANN model compared with other models, such as SVM, MLR, and regression trees.

## 4. Conclusions

The objective of this study was to streamline the process of predicting the compressive strength of concrete using various data-driven models, such as artificial neural networks (ANN), support vector machines (SVM), multiple linear regression (MLR), and regression trees. These methods were compared based on four different efficiency metrics. MATLAB software was employed for all calculations related to data-driven models due to its user-friendly interface, eliminating the need for advanced programming skills. The software’s intuitive design allows users to become familiar with basic tools and execute program steps sequentially.

This study utilized eight different variables to forecast the compressive strength of concrete, all of which are fundamental requirements in concrete manufacturing. In cases where certain items were not used in concrete production, a null value was employed in the prediction model. The results from all models demonstrated that the ANN model outperformed the other three models. This indicates that predicting the compressive strength of concrete can be more accurate and efficient using the ANN method, reducing the waiting period to determine concrete strength. By incorporating material weights during concrete production, the model provides strength results simultaneously with production, eliminating the need for specific equations or problem-solving methods.

The unique feature of retraining data and learning makes the ANN more suitable for prediction compared with other models. This study conducted an extensive comparison of the ANN model with three popular data prediction models, highlighting its importance in achieving high-accuracy results. Additionally, this study offers a clear explanation of all parameters used in the ANN model, facilitating easy comprehension for future predictions.

## Figures and Tables

**Table 1:** Dataset parameters and statistical value.

Parameter	Unit	Mean	Maximum	Minimum	Standard Deviation
Cement	kg/m^3^	281.166	540	102	104.507
Blast furnace slag	kg/m^3^	73.895	359.4	0	86.279
Fly ash	kg/m^3^	54.187	200.1	0	63.996
Water	kg/m^3^	181.566	247	121.75	21.356
Superplasticizer	kg/m^3^	6.203	32.2	0	5.973
Coarse aggregate	kg/m^3^	972.919	1145	801	77.754
Fine aggregate	kg/m^3^	773.579	992.6	594	80.175
Age	Day	45.662	365	1	63.170
Concrete compressive strength	MPa	35.818	82.599	2.332	16.706

**Table 2:** Variance of different attributes.

Attributes	Variance
Cement	10,921.74
Blast furnace slag	7444.084
Fly ash	4095.548
Water	456.0602
Superplasticizer	35.6826
Coarse aggregate	6045.656
Fine aggregate	6428.099
Age	3990.438

**Table 3:** Correlation table.

Correlational Coefficient	Compressive Strength
Cement	0.498
Blast furnace slag	0.135
Fly ash	−0.106
Water	−0.29
Superplasticizer	0.366
Coarse aggregate	−0.165
Fine aggregate	−0.167
Age	0.329
Compressive strength	1

**Table 4:** Different ANN architecture models and their performance.

Architecture	Value of R			
	Train	Validation	Test	All
8-10-1	0.96	0.91	0.93	0.95
8-20-1	0.96	0.92	0.93	0.95
8-15-1	0.96	0.96	0.97	0.96
8-20-10-1	0.97	0.94	0.87	0.95

**Table 5:** Optimum parameters for the best-trained model.

Network Properties	
Parameter	Value
Network type	Feed-forward backprop
Training function	TRAINLM
Adaption learning function	LEARNGDM
Performance function	MSE
No. of layers	2(1-TANSIG, 2-TANSIG)
Number of neurons	(1st Layer-15, 2nd layer-10)
Data division	Random
Training	Levenberg–Marquardt
Performance	Mean square error
Calculation	MEX

**Table 6:** Performance measurement of MLR training data.

Model	Training			
	MAD	MSE	RMSE	MAPE
MLR	3.6159	48.157	6.9395	13.2042

**Table 7:** Result of different SVM-trained models.

SVM Model	RMSE
Linear SVM	10.966
Quadratic SVM	8.1616
Cubic SVM	6.9287
Fine Gaussian SVM	10.241
Medium Gaussian SVM	7.1213
Coarse Gaussian SVM	9.168

**Table 8:** Result of different regression tree-trained models.

Tree Model	RMSE
Fine tree	7.5767
Medium tree	8.2006
Coarse tree	10.083

**Table 9:** Performance measurement of different models.

Model	Simulation			
	MAD	CC	RMSE	MAPE
Artificial neural network (ANN)	4.443	0.712	6.092	15.112
Multiple linear regression (MLR)	8.1824	−0.57	10.45	32.51
Support vector machine (SVM)	4.5813	0.724	6.3121	15.9816
Regression tree	4.6908	0.641	6.6029	17.6101

## Data Availability

Data download link contained within the article.
